# Developmental expression and function of *DKKL1/Dkkl1* in humans and mice

**DOI:** 10.1186/1477-7827-10-51

**Published:** 2012-07-21

**Authors:** Qiuxia Yan, Xiaoping Wu, Cairong Chen, Ruiying Diao, Yongqing Lai, Jun Huang, Jing Chen, Zhou Yu, Yaoting Gui, Aifa Tang, Zhiming Cai

**Affiliations:** 1 Center for Reproductive Medicine, Department of Obstetrics and Gynecology, The People's Hospital of Qingyuan, The Fifth Affiliated Hospital of Medical College of Jinan University, Qingyuan, China; 2 Guangdong Key Lab of Male Reproductive Medicine and Genetics, Peking University Shenzhen Hospital, Shenzhen, China; 3 Shenzhen Second People's Hospital, The First Affiliated Hospital of Shenzhen University, Shenzhen, China; 4 Institute of Tissue Transplantation and Immunology, Jinan University, Guangzhou, China

**Keywords:** *DKKL1/Dkkl1*, Affymetrix Genechip, Testis, Spermatogenesis

## Abstract

**Background:**

Experiments were designed to identify the developmental expression and function of the Dickkopf-Like1 (*DKKL1/Dkkl1*) gene in humans and mice.

**Methods:**

Mouse testes cDNA samples were collected at multiple postnatal times (days 4, 9, 18, 35, and 54, as well as at 6 months) and hybridized to Affymetrix mouse whole genome Genechips. To further characterize the homologous gene *DKKL1* in human beings, the expression profiles between human adult testis and foetal testis were compared using Affymetrix human Genechips. The characteristics of *DKKL1/Dkkl1* were analysed using various cellular and molecular biotechnologies.

**Results:**

The expression of *Dkkl1* was not detected in mouse testes on days 4 or 9, but was present on days 18, 35, and 54, as well as at 6 months, which was confirmed by RT-PCR and Western blot results. Examination of the tissue distribution of *Dkkl1* demonstrated that while *Dkkl1* mRNA was abundantly expressed in testes, little to no expression of *Dkkl1* was observed in the epididymis or other tissues. In an *in vitro* fertilization assay, a *Dkkl1* antibody was found to significantly reduce fertilization. Human Genechips results showed that the hybridization signal intensity of *DKKL1* was 405.56-fold higher in adult testis than in foetal testis. RT-PCR analysis of multiple human tissues indicated that *DKKL1* mRNA was exclusively expressed in the testis. Western blot analysis also demonstrated that *DKKL1* was mainly expressed in human testis with a molecular weight of approximately 34 kDa. Additionally, immunohistochemical staining showed that the *DKKL1* protein was predominantly located in spermatocytes and round spermatids in human testes. An examination of the expression levels of *DKKL1* in infertile male patients revealed that while no *DKKL1* appeared in the testes of patients with Sertoli cell only syndrome (SCOS) or cryptorchidism, *DKKL1* was observed with variable expression in patients with spermatogenic arrest.

**Conclusions:**

These results, together with previous studies, suggest that *DKKL1/Dkkl1* may play an important role in testicular development and spermatogenesis and may be an important factor in male infertility.

## Background

Spermatogenesis is characterized by successive periods of regulated cell proliferation, meiosis, and haploid differentiation. Abnormalies during any step of spermatogenesis could cause male infertility. It is estimated that approximately 2,000 genes regulate the process of spermatogenesis, and most of these genes are present on the autosomes, while approximately 30 genes are found on the Y chromosome [1]. Recent studies have shown that *Septin12*[2], *Fank1*[3],*CKT2*[4], *RGS22*[5] and *NANOS2*[6] are specifically expressed in the testis and are functionally involved in spermatogenesis. Identification of these genes and studies on their spatial and chronological expression patterns are essential for understanding the mechanisms of spermatogenesis and male infertility [7-9].

Recently, using Affymetrix Genechips, we identified 2,058 up-regulated transcripts during the developmental period from postnatal day 4 to 6 months in mice [10] Among these transcripts were 292 testis-specific genes [11], including *TSG23*[12], *TSC21*[13], *TSC24*[14] and *TSC77*[15]. In the present study, another gene, Dickkopf-Like1 (*DKKL1/Dkkl1*) was identified using Affymetrix mouse and human Genechips.

*DKKL1/Dkkl1* was identified independently as a distant homologue to the Dickkopf (Dkk) family of proteins that modulate WNT/β-catenin signalling [16]. In contrast to conventional Dkks, Dkkl1 does not modulate WNT/β-catenin canonical signalling [17]. Several reports have concluded that *Dkkl1* mRNA is expressed at high levels in adult mice testis in the spermatogenic epithelium of the seminiferous tubules [18] and in developing spermatocytes where *Dkkl1* accumulates first in developing acrosomes and then in the acrosome of mature sperm [19]. This suggests that *Dkkl1* may play a role in spermatocyte development and maturation in mice. However, little is known about the character and function of *DKKL1* in human testes. Therefore, the present study was set out to explore the spatial and chronological expression of *DKKL1/Dkkl1* in human and mouse testes and to compare the mRNA and protein expression levels of *DKKL1/Dkkl1* in fertile and infertile human testes. A clearer understanding of the role of *DKKL1/Dkkl1* in testes may help elucidate the biological principles underlying the increasing rate of male infertility and may provide targets for the development of a male contraceptive.

## Methods

### Sources of samples

Male and female Balb/c mice were obtained from the Animal Laboratory Centre of South Medical University (Guangzhou, China) and maintained in a temperature and humidity-controlled room. All animals had free access to standard mouse chow and water. Male and female mice (1:3) were mated naturally, and the day of birth was designated as day 1. Testes were individually collected from Balb/c mice on days 4, 9, 18, 35, and 54, as well as at 6 months (m 6). Testis samples at postnatal days 4 (n = 30), 9 (n = 20), 18 (n = 15), 35 (n = 8), and 54 (n = 4), as well as at m 6 were collected. Other organs including the brain, heart, liver, spleen, lung, kidney, muscle, stomach, intestine, bladder and epididymis were also collected from adult mice (n = 4).

Testis biopsy material from male infertility patients aged 20–40 years with Sertoli cell only syndrome, cryptorchidism or spermatogenic arrest were obtained from Peking University Shenzhen Hospital, Shenzhen, China. A sample of fertile human testis was obtained from an adult male patient (aged 27 yr) undergoing bilateral orchiectomy for the treatment of prostate carcinoma, and a sample of foetal testis was obtained from a naturally aborted embryo (aged 6 m). In addition, human tissues, including ovary, kidney, uterus, prostate, thyroidea, stomach and oesophagus, were also collected. All samples were frozen in liquid nitrogen and then immediately stored at −80°C. All patients signed consent forms approved by the Committee on Human Rights in Research of the Ethics Committee at Peking University Shenzhen Hospital, Shenzhen, China. Animal experiments were approved by the Animal Test Centre of China.

### cDNA microarray hybridization

The screen for *Dkkl1* was undertaken by hybridizing cDNA from mouse testes at six developmental stages with commercially available Affymetrix mouse Genechips, which contain 45,000 pairs of probes including 39,000 transcripts, as previously described [10]. The homologous human gene, *DKKL1*, was also screened for by comparing the expression profiles of human adult and foetal testis using Affymetrix human Genechips containing 47,000 transcripts derived from approximately 38,500 well-substantiated human genes. All of these procedures were carried out as described by Affymetrix. After hybridization, the array was washed, stained with streptavidin phycoerythrin using the Affymetrix Genechip Fluidics Workstation 400, and scanned on a Hewlett-Packard gene array scanner (Hewlett-Packard Co., Palo Alto, CA, USA). After the arrays were scanned, the signals generated were quantified and analyzed using MAS 5.0 software. Absolute and comparison analyses were also performed using MAS 5.0. After normalization of these data, experimental arrays were compared with baseline arrays to detect changes in the expression of transcripts across samples targeted to different arrays (see http://www.Affymetrix.com for details on the statistics of these analyzis).

### Semi-quantitative RT-PCR

Semi-quantitative RT-PCR was performed to analyse and confirm the expression of the *DKKL1/Dkkl1* genes. Total RNA (2 μg) was reverse-transcribed into cDNA in a reaction primed by an oligodeoxynucleotide (dT)_18_ primer using RevertAid^TM^M-Mulv Reverse Transcriptase (Fermentas, Glen Burnie, MD, USA) according to the manufacturer’s instructions. Polymerase chain reaction (PCR) primers for *DKKL1/Dkkl1*, *β-actin* and *GAPDH* were synthesized by Shanghai Bioengineering Inc. (Shanghai, China; Table 1). The PCR reaction was initiated by hot start at 94°C for 4 min, followed by 33 cycles of 94°C for 30 s, 64°C for 30 s and 72°C for 40 s, followed by extension at 72°C for 5 min. PCR products were run out on a 1% agarose gel in 0.5x TBE buffer (30 min at 100 V) and analyzed using a Rapid Agarose Gel Electrophoresis System (Wealtec Corp.,Sparks, NE, USA).

**Table 1:** Oligonucleotide sequences used in RT-PCR analysis

**Transcripts**	**Annealing Temperature (°C)**	**Product size (bp)**	**Sequence direction (5’-3’)**
*DKKL1*	58	299	Sense: TGCTGCTCCTCTCTACCCT
			Antisense: CTCTCCTGTCTTGTTGTCGG
*Dkkl1*	55	217	Sense: TCGTGTCCTCCTCTGCTCTCT
			Antisense: TTGCCCATTCTGTGCTCCT
*β-actin*	55	281	Sense: AACAGTCCGCCTAGAAGCAC
			Antisense: CGTTGACATCCGTAAAGACC
*GAPDH*	58	100	Sense: GCTCTCTGCTCCTCCTGTTC
			Antisense: GACTCCGACCTTCACCTTCC

### *DKKL1* transcription analysis in the testes of patients with male infertility

Testicular tissues were obtained via biopsy from 15 patients with male infertility at the Peking University Shenzhen Hospital (Shenzhen, China). The clinical diagnoses based on testicular biopsy were Sertoli-cell-only syndrome (SCOS), cryptorchidism, and spermatogenic arrest at different stages. Total RNA (about 2 μg) was extracted using TRIzol (Invitrogen, Carlsbad, CA, USA). Reverse transcription and PCR were performed as described above.

### Protein extraction and Western blot analysis

Human and mouse tissues were lysed with lysis buffer in the presence of a protease inhibitor cocktail (Merck, USA) and kept on ice for 1 h. After centrifugation at 12,000 g for 20 min at 4°C, the resulting supernatant was collected for Western blot analysis. After the protein concentration was determined by BCA protein assay (Thermo Fisher Scientific Inc., USA), supernatant fractions from the lysate were mixed with 6x SDS sample buffer and boiled for 10 min. Samples were reduced with 5% β-mercaptoethanol and stored at −20°C until used.

Extract samples containing approximately 30 μg protein were separated by 10% SDS-polyacrylamide gel electrophoresis (SDS-PAGE), and the extracts were then transferred onto polyvinylidene difluoride (PVDF) membranes (MilliPore, Bedford, MA, USA). The membranes were blocked in TBST (5 mmol Tris–HCl, pH 7.4; 136 mmol NaCl; and 0.05% Tween20) containing 2% BSA overnight at 4°C. The next day, the membranes were hybridized at room temperature for 4 h with rabbit anti- *DKKL1/Dkkl1* antibody (ABGENT, USA) at a dilution of 1 μg/ml and rabbit anti-GAPDH (Abcam) as an internal control, followed by three washes for 10 min with TBST. Next, the blots were incubated for 1 h at room temperature with HRP-conjugated goat anti-IgG rabbit antibody (1:5000; Abcam), and washed three times with TBST. Bound antibodies were detected by electro-chemiluminescence using SuperSignal West Dura substrates (Pierce Biotechnology Inc.) according to the manufacturer's recommendations and visualized by fluorescence detection equipment (ChemiDoc XRS, BIO-RAD, Hercules, CA, USA).

### In vitro fertilization (IVF)

Female mice were superovulated, and the stage MII oocytes were collected from mice oviducts, as described [20]. Mouse spermatozoa from cauda epididymis were capacitated in Human Tubal Fluid medium (HTF; In-Vitro Fertilization. Inc., USA) within 30 min, and the spermatozoa (in drops of 50 μl with a concentration of 5 × 10^4^/ml) were incubated for 30 min with either HTF or HTF containing anti- *Dkkl1* antibody at a dilution of 1 μg/ml. The treated spermatozoa (50 μl) were deposited into Cleavage medium (In-Vitro Fertilization. Inc., USA) containing 30–35 mouse oocytes and incubated for 2 h. The unbound spermatozoa were washed away. To analyze the IVF rate, two pronuclear cells were examined 6 h after fertilization. The zygote cleavages were counted at 42 h [21].

### Immunohistochemistry

The specimens were fixed for 4 hr in 10% formalin and then embedded in paraffin, sectioned at 5 μm, and mounted on silane-coated slides. For immunohistochemistry, sections were dewaxed and rehydrated through descending grades of alcohol to distilled water, followed by incubation in 2% hydrogen peroxide to quench the endogenous peroxidase activity and then washed in PBS. Subsequently, nonspecific binding was blocked with goat serum (Fuzhou Maixin Biotechnology, China) for 2 h, followed by incubation with polyclonal rabbit anti- *DKKL1/Dkkl1* antibody (ABGENT, USA) overnight at 4°C. Following three washes in PBS, the sections were incubated with horseradish peroxidase (HRP) conjugated goat anti-rabbit secondary antibody (Fuzhou Maixin Biotechnology, China) for 1 h at room temperature. Immunoreactive sites were visualized with diaminobenzidine (DAB) and mounted for bright field microscopy (DMLB; Leica Microsystems, Germany). Negative control sections were incubated with the buffer 1% BSA in place of the primary antibody.

## Results

### The expression patterns of *DKKL1/Dkkl1* as shown by Genechip analysis

**Figure 1:**
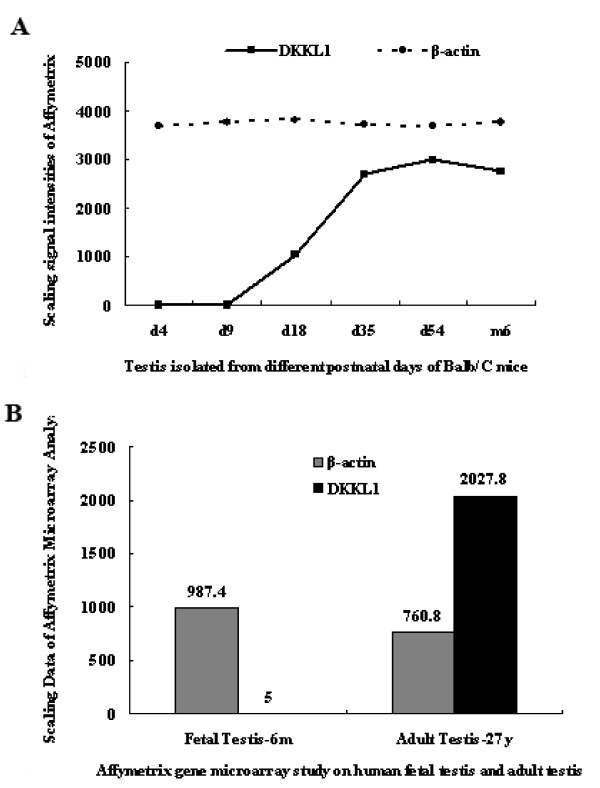
**Developmental expression pattern of*DKKL1/Dkkl1*during spermatogenesis detected by Affymetrix chip analysis.A**: Mice testis was isolated from postnatal Balb/C mice on days 4, 9, 18, 35, and 54, as well as on 6 months and applied to whole genomic analysis by Affymetrix chip. The scaling signal intensities of *Dkkl1* from mouse testis on days 4, 9, 18, 35, and 54, as well as on 6 months were 1.8, 9.9, 1030.5, 2696.8, 2987.9 and 2752.7, respectively. Signals on day 4 and 9 were not detected. On the other hand, the signal intensities of *β-actin* were 3688.8, 3764.78, 3812.9, 3696.87, 3679.71 and 3757.12, respectively. **B**: Hybridization using a human Affymetrix Genechip revealed differential expression of *DKKL1* in human foetal (6 months) and adult (27 yr) testes. The hybridization intensity of *DKKL1* in foetal and adult testes was 5 and 2,027.8, respectively, and the signal intensity of *β-actin* was 987.4 and 760.8, respectively.

By hybridizing mouse testes of six different developmental stages with commercially available Affymetrix mouse Genechips, we identified an age-dependent gene, *Dkkl1* (GenBank accession number AF_177399). The hybridization signal intensities from the testes of Balb/c mice on postnatal days 4, 9, 18, 35, and 54 as well as on 6 months were 1.8 (absent, or no expression on the chip, A), 9.9 (A), 1,030.5 (present, or expression on the chip, P), 2,696.8 (P), 2,987.9 (P) and 2,752.7 (P), respectively. That is, by Affymetrix chip analysis, the signal on days 4 and 9 was not detectable but after day 18 it gradually increased as the development of the mouse testes progressed. In comparison, the signal intensities of *β-actin* were 3,688.88, 3,764.78, 3,812.9, 3,696.87, 3,679.71, and 3,757.12, respectively (Figure 1A). The homologous human gene *DKKL1* (GenBank accession number NM_014419) was observed by hybridizing human adult or foetal testis cDNA samples with a human Affymetrix Genechip. This gene was more highly expressed in adult testis than in foetal testis. The hybridization signal intensity was 2,027.8 in adult and 5 in foetal testis, with an expression level in the adult testis approximately 405.56-fold higher than that in the foetal testis. The signal intensities of *β-actin* were 987.4 and 760.8, respectively (Figure 1B).

### Expression profile of *Dkkl1* in mice

**Figure 2:**
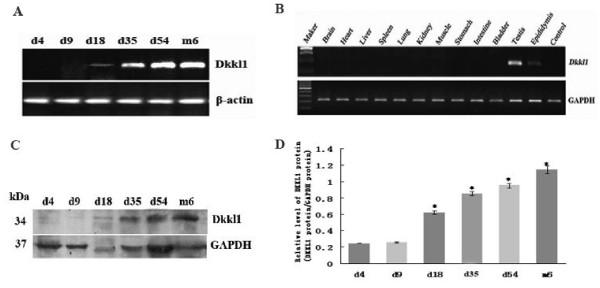
**Expression pattern of*Dkkl1*in mice**. **A**: Mouse *Dkkl1* mRNA was not expressed in mouse testis on days 4 and 9 and was weakly expressed on day 18. The expression increased gradually from day 18 to 54 and remained stable after day 54. *β-actin* was used as an internal loading control. **B**: The expression pattern of *Dkkl1* mRNA in 12 different mouse tissues is shown. Except for a trace amount of *Dkkl1* mRNA in the epididymis, expression of *Dkkl1* was found only in testis. *GAPDH* was used as an internal control. **C**: Representative Western blot analyses of protein from samples obtained from testes at postnatal days 4, 9, 18, 35, and 54, as well as at 6 months. The expression of *GAPDH* was used as an internal standard for normalization. The protein level of *Dkkl1* increased during testicular development, which is consistent with the expression of *Dkkl1* mRNA. The size of the *Dkkl1* protein was approximately 34 kDa. **D**: In each of three replicate analyses, Western blot results were quantified, and the results were expressed as the ratio of *Dkkl1*/*GAPDH*. The bars represent the mean ± SD of the data for each age, and the bars marked with asterisks showed statistically significant differences (p < 0.05).

To authenticate the expression profile of *Dkkl1* during the development of the mouse testes, we performed RT-PCR and Western blot analysis using mice testes obtained at different postnatal developmental stages. Expression of *Dkkl1* was detected after day 18 and gradually increased from day 18 to 54 (Figure 2A). Furthermore, he levels of *Dkkl1* protein increased during testicular development, which is consistent with the expression of *Dkkl1* mRNA (Figure 2C). The results from RT-PCR and Western blot analysis were consistent with our Affymetrix chip analysis, which suggests that the expression profile of *Dkkl1* is developmental stage specific. The distribution of *Dkkl1* was examined using multi-tissue RT-PCR in 12 different mouse tissues including brain, heart, liver, spleen, lung, kidney, muscle, stomach, intestine, bladder, testis, and epididymis. The gene was expressed at high levels in testis and at weak levels in epididymis, and was not found in the other tissues (Figure 2B).

### In vitro fertilization was reduced by *Dkkl1* antibody

**Figure 3:**
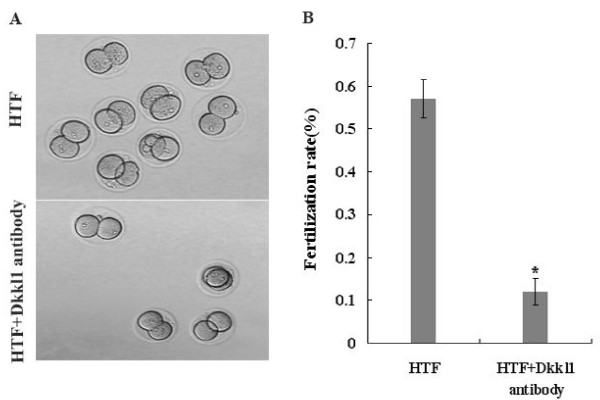
**The inhibitory effect of*Dkkl1*antibody on*in vitro*fertilization**. Successful fertilization was assayed by zygote cleavage. **A**, Top image: spermatozoa incubated with HTF demonstrated successful fertilization; Bottom image: spermatozoa incubated with HTF + *Dkkl1* antibody demonstrated unsuccessful fertilization. **B**: The bars represent the mean ± SD of three replicate analyses, and bars marked with asterisks showed statistically significant differences (p < 0.05).

We used in vitro fertilization assays to investigate a possible role for *Dkkl1* in fertilization. Successful in vitro fertilization was identified by the appearance of embryos at the 2-cell and 4-cell stages (Figure 3A). While the fertilization rate of the HTF group exceeded 57%, the fertilization rate of the HTF *+ Dkkl1* antibody group dropped significantly to 12% (Figure 3B). This supports the notion that *Dkkl1* plays a role in fertilization and that the *Dkkl1* antibody employed in this study can block its action.

### Tissue distribution of *DKKL1* mRNA and protein in humans

**Figure 4:**
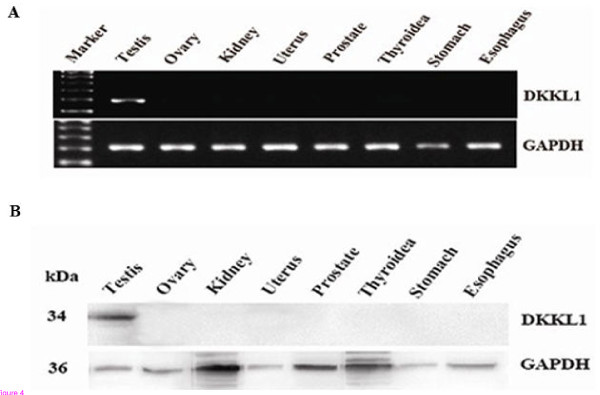
**Expression pattern of*DKKL1*mRNA and protein in humans**. **A**: Examination of the tissue distribution of *DKKL1* mRNA demonstrated that it was strongly expressed in testis and not expressed in 7 other organs. *GAPDH* was used as a loading control. **B**: Human tissues were subjected to Western blot analysis with antibodies against *DKKL1*. The *DKKL1* antibody recognized a band at approximately 34 kDa. This protein was predominantly expressed in testis. *GAPDH* was used as a loading control.

The expression profile of *DKKL1* in various tissues was also studied using multi-tissue RT-PCR. Of the 8 human organs tested (testis, ovary, kidney, uterus, prostate, thyroidea, stomach and oesophagus), *DKKL1* was exclusively expressed in the testis (Figure 4A). To examine the specificity of the *DKKL1* antibody and confirm the results of the RT-PCR analysis, Western blot analysis was carried out on the same tissue samples. The antibody recognized a distinct band at 34 kDa, which is comparable to the predicted molecular weight of *DKKL1*. The band was only detected in human testis, suggesting that *DKKL1* is primarily expressed in human testis (Figure 4B).

### Abnormal expression of *DKKL1* mRNA in the testes of patients with male infertility

**Figure 5:**
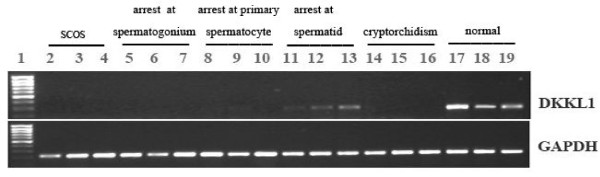
**Abnormal expression of*DKKL1*mRNA in the testes of patients with male infertility.** Top: RT-PCR studies examined *DKKL1* expression in 15 infertile patients with SCOS (lanes 2–4), with spermatogenic arrest at various stages (lanes 5–7, arrest at spermatogonium; lanes 8–10, arrest at primary spermatocyte; lanes 11–13, arrest at spermatid), with cryptorchidism (lanes 14–16), or with normal testis(lanes 17–19). The results indicate that *DKKL1* is not expressed in the testes of patients with SCOS or cryptorchidism. *DKKL1* expression is variable in patients with spermatogenic arrest. In patients with arrest at the spermatogonium and primary spermatocyte stage, *DKKL1* is not expressed, but in patients with arrest at the spermatid stage, *DKKL1* is weakly expressed. In normal samples containing spermatogenic cells of every stage, *DKKL1* expression is strong. Bottom: The expression of human *GAPDH* mRNA is displayed as a positive control.

To investigate the contribution of *DKKL1* to spermatogenesis, we examined its expression in the testes of fertile and infertile men. RT-PCR results indicated that *DKKL1* was not expressed in the testes of patients with either SCOS or cryptorchidism. *DKKL1* expression in patients with spermatogenic arrest varied. In patients with arrest at the spermatogonium and primary spermatocyte stages, *DKKL1* expression was not detected; however, in patients with arrest at the spermatid stage, *DKKL1* expression levels were weak or absent. In fertile men with spermatogenic cells of every stage, the *DKKL1* expression level was high. These results indicate a trend of increasing *DKKL1*expression as spermatogenic cells mature. The expression of GAPDH was comparable in the testes of all samples (Figure 5).

### Expression of *DKKL1* protein in the testis of infertile patients

**Figure 6:**
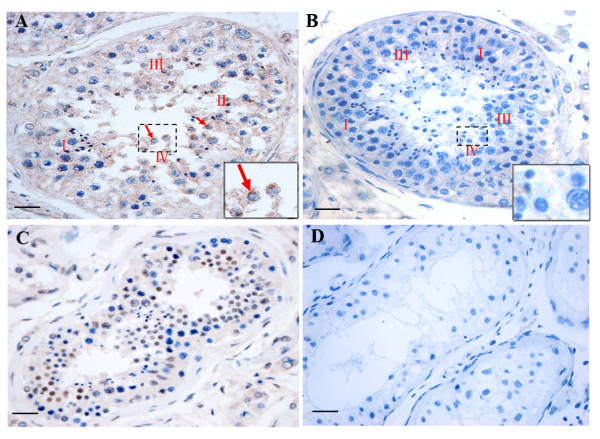
**Localization and expression characteristics of*DKKL1*protein in the testis of fertile and infertile patients by immunohistochemistry assay.A**: *DKKL1* protein was predominantly located in the spermatocytes and round spermatids in fertile testes. **B**: No staining was observed in tissue sections when the *DKKL1* antibody was replaced by buffer containing 1% BSA. **C**: In the spermatogenic arrest testis, the layers of spermatogenesis cells decreased and a number of vacuoles were observed in the lumen. Reduced *DKKL1* protein signal was detected in spermatocytes and round spermatids. **D**: In the SCOS or cryptorchidism testis, the basal membrane of the seminiferous tubules was thickened; only Sertoli cells and spermatogonial cells were found in the seminiferous epithelia. No *DKKL1* protein signal was detected. Scale bar = 25 μm.

In normal testis, all stages of spermatogenic cells were found to be present in the seminiferous epithelia. *DKKL1* protein was predominantly located in the spermatocytes and round spermatids and was not found to be located in Leydig cells or basal membranes. Following the method of Clermonts as cited in Amann’s review [22], we determined the spermatogenic stages present in the human testis samples. Further analysis indicated that intense *DKKL1* localization was observed in stages II, III and IV of spermatogenesis, whereas it was lower in stage I (Figure 6A). In the testis from patients with spermatogenic arrest, the expression of *DKKL1* protein was significantly decreased in spermatocytes and round spermatids (Figure 6C). No *DKKL1* protein signal was detected in testis of patients with SCOS or cryptorchidism (Figure 6D).

## Discussion

It has been previously shown that spermatogenesis is mainly regulated by testis-specific gene activation. Investigation of testis-specific genes is expected to lead to a broader and more thorough understanding of spermatogenesis. Many genes related to human and mouse spermatogenesis have been identified by our previous research [12-15]. The present study focuses on the characterization of a newly recognized gene, *DKKL1/Dkkl1*.

As spermatogenesis is divided into three major phases, namely proliferation and differentiation of spermatogonia, meiosis and spermiogenesis [23], the expression patterns of *Dkkl1* in mouse testes at different developmental stages were first investigated using a gene chip approach. The six selected developmental stages represent the major stages of germ cell development during the first wave of spermatogenesis: day 4, cells with stem cell properties; day 9, spermatogonia mitosis; day 18, spermatocyte meiosis; day 35, round spermatid production and elongated spermatid formation, also called spermiogenesis; day 54, normal postpubertal spermatogenesis; 6 months, elongated spermatids and immature sperm [24]. As demonstrated in the mouse gene chip results, the expression of *Dkkl1* was detected on days 18, 35, and 54, as well as at 6 months, but it was not detected on days 4 and 9. This was further verified by RT-PCR and Western blot analysis. Analysis revealed that *Dkkl1* was weakly expressed in mouse testis on day 18, with expression increasing after day 18 and remaining stable after day 54. Based on the expression characteristics of *Dkkl1* in mice, we suggest that the expression of *Dkkl1* mRNA and protein are associated with the postmeiotic phase of spermatogenesis and with the generation of late pachytene spermatocytes and round spermatids. In addition, the results of multi-tissue RT-PCR showed that this gene was highly expressed in testis and weakly expressed in the epididymis. A possible reason for the weak expression of *Dkkl1* in the epididymis of mice is that some immature and mature sperm are stagnated in the epididymis.

Although *Dkkl1* has been previously suggested to be important for male fertility i*n vitro*[25], surprisingly, *Dkkl1*−/−mice are not only viable and fertile, but both male and female Dkkl1−/− mice produce offspring at efficiencies comparable to wild-type animals [17,26]. These studies made it impossible to evaluate the contribution of *Dkkl1*. So what role might *Dkkl1* play in fertilization? In the present study, we used an *in vitro* fertilization assay to investigate the role of *Dkkl1*. Following the use of a *Dkkl1* neutralizing antibody, the fertilization rate was significantly reduced. This result is consistent with a previous study [25] that found that *Dkkl1* is required for efficient fertilization *in vitro*. It is likely that *Dkkl1* plays a role in the penetration of spermatozoa into oocytes, and during this time window, the neutralizing antibody has a chance to block its action. However, IVF provides an assay for detecting fertilization-related problems that are not apparent in vivo. More likely is the possibility that a delay in fertilization caused by the absence of *Dkkl1* is compensated by other factors during spermatogenesis or preimplantation development.

Given that *Dkkl1* was closely linked to mouse spermatogenesis, what is its relationship to human spermatogenesis? To address this question, we next conducted hybridization of adult and foetal testes samples to a human gene chip. The results indicated that *DKKL1* was expressed at a higher level in adult testis than in foetal testis. The relative hybridization signal intensity of *DKKL1* in adult testis was 405.56 times that in foetal testis. There are only Sertoli cells and undifferentiated spermatogonia cells in the seminiferous tubules of the foetal testis, whereas the seminiferous tubules of the adult testis contain not only Sertoli cells and spermatogenous cells, but also various spermatogenic cells. In other words, there are many developmental stages of germ cells represented in adult testis that are not found in foetal testis. Many genes expressed in the testis are developmental stage specific or cell type specific, which reflects the demands of tissue development [27]. It has been demonstrated that other genes with differential expression between adult and foetal testis, namely *Spef1*[28], *Akap4*[29], and *Rosbin*[30], are spermatogenesis-specific. Thus, the results of our gene chips analysis provided an important clue that *DKKL1* might be associated with testis development and spermatogenesis in humans.

Whether the expression of testis development and spermatogenesis genes were altered in male infertility? Previous study have shown that patients with SCOS or spermatogenesis arrest at spermatocyte do not express a novel human testis-specific and spermatogenesis protein NYD-SP12 [31]. Recently, it has been reported that the signal of the human testis developmental gene *SPATA12* was not detected in patients with cryptorchidism or SCOS [32]. To further validate the function and role of *DKKL1* in male infertility, we examined the *DKKL1* mRNA transcript levels as well as protein levels among the patients with SCOS, cryptorchidism or spermatogenic arrest. Most of them had no or insufficient expression of *DKKL1* protein in the testes. Only the control fertile male testis sample, in which every stage of spermatogenic cell was represented, fully expressed *DKKL1*. The trend of increasing expression with the presence of the more mature spermatogenic cells in testis is consistent with the development-dependent characteristics of *DKKL1* mRNA. It was revealed that decreased expression of *DKKL1* was associated with spermatogenic failure in infertile men.

## Conclusions

We have provided evidence that *DKKL1/Dkkl1* is potentially involved in human and mouse spermatogenesis. Further investigation of molecular mechanisms, such as the distribution of *DKKL1* in multiple tissues by *in situ* hybridization or immunohistochemical staining, and its interaction with other proteins by immunoprecipitation or the yeast two-hybrid system, is required to determine its biological function in mammalian spermatogenesis. These studies are currently under way. Moreover, the screening of *DKKL1* gene mutations in patients with SCOS, cryptorchidism and spermatogenic arrest by direct sequencing may help us to understand the role of *DKKL1* in clinical male infertility.

## Competing interests

The authors declare that they have no competing interests.

## Authors’ contributions

QY, XW and CC participated in the design of the study, collected the materials, and carried out all experiments. QY drafted the manuscript. RD, YL, JH, JC and ZY collected the materials and helped to carry out the cDNA microarray hybridization, RT-PCR, immunohistochemistry, and Western blot analyses. AT, YG and ZC conceived of the study, participated in its design and coordination and helped to draft the manuscript. All authors read and approved the final manuscript.
